# Hemi-Percutaneous Epiphysiodesis Using Transphyseal Screws at Lateral Proximal Tibias After Epiphyseal Fusion of Distal Phalanges in the Hand Results in Undercorrection of Genu Varum

**DOI:** 10.3390/medicina60111818

**Published:** 2024-11-05

**Authors:** Kyung Rae Ko, Jae Woo Shim, Jong Sup Shim, Dong Suk Kim, Soonchul Lee

**Affiliations:** 1 Department of Orthopedic Surgery, Samsung Medical Center, Sungkyunkwan University School of Medicine, Seoul 06351, Republic of Korea; krmd.ko@gmail.com (K.R.K.); osdrshim@gmail.com (J.W.S.); arr9127@hanmail.net (D.S.K.); 2 Department of Orthopedic Surgery, Knot Hospital, Suwon-si 16687, Gyeonggi-do, Republic of Korea; jss3505@skku.edu; 3 Department of Orthopedic Surgery, CHA Bundang Medical Center, CHA University School of Medicine, Seongnam-si 13488, Gyeonggi-do, Republic of Korea

**Keywords:** coronal plane alignment, genu varum, tibia vara, epiphysiodesis, hemiepiphysiodesis, transphyseal screw, PETS, hemi-PETS

## Abstract

*Background and Objectives*: To investigate postoperative courses after hemi-percutaneous epiphysiodesis using transphyseal screws (PETS) for genu varum. We especially focused on the degree of skeletal maturation that results in undercorrection. *Materials and Methods*: We identified patients with idiopathic genu varum treated with hemi-PETS at the proximal tibia and followed-up to the completion of skeletal maturation. The acceptable correction was defined as the (1) final deformity < varus 1.0° or (2) final correction angle obtained by surgery (f-CA) > mean value of preoperative deformity. *Results*: In our cohort of 29 patients and their 29 lower limbs (one side was randomly selected in bilateral cases), the mean hip–knee–ankle (HKA) alignment was varus 6.5 ± 1.3° at the time of hemi-PETS. The mean f-CA was 5.8 ± 2.0° with a rebound of 0.3 ± 1.0°. Ten patients showed the finding of partial or complete fusion of the epiphysis of distal phalanges in the hand at the time of hemi-PETS (the fusion group, FG). Their f-CA was 4.0 ± 1.9° (with preoperative deformity of 6.9 ± 1.4°), which was significantly smaller than that (6.7 ± 1.3°, *p* = 0.001) of remaining 19 patients (the open group, OG). The acceptable correction was obtained in all 19 patients of the OG. Otherwise, it was obtained in two patients in the FG (*p* < 0.001). The other two patients in the FG preoperatively showed a complete epiphyseal fusion of the distal phalanges in the hand, and their f-CA was 0.7 and 1.1°, respectively. *Conclusions*: The degree of skeletal maturation corresponding to epiphyseal fusion of distal phalanges in the hand results in undercorrection after the hemi-PETS performed at the proximal tibia for genu varum.

## 1. Introduction

Genu varum is not rare in adolescents. Its prevalence was reported as 11.4% in a large cohort of healthy adolescents [1]. Furthermore, there is a great need to pay attention to this topic, since recent studies suggest that a relation exists between sports participation in adolescents and its development [2,3,4]. Genu varum is not just a cosmetic concern, as the malalignment of the lower limb can increase the risk of degenerative changes in the knee joint [5]. Unlike in adults with angular deformity, who can be treated with an osteotomy, angular deformity in growing patients can be corrected by hemiepiphysiodesis with less morbidity [6,7,8,9,10,11,12].

After performing hemiepiphysiodesis, which inhibits a certain portion of the physis, gradual correction of angular deformity comes into effect by the growth of the opposite portion. Thus, a certain period of remaining growth is necessary to obtain sufficient corrections, and surgery performed very near to the completion of skeletal maturation can result in undercorrection. Among various options for hemiepiphysiodesis, using transphyseal screws (i.e., hemi-percutaneous epiphysiodesis using transphyseal screws, hemi-PETS) is minimally invasive and results in less pain and shorter hospital stays [12]. However, in terms of determining the timing of surgery, the delayed effect of PETS [13,14,15] makes it difficult to calculate the minimum period of remaining growth for sufficient correction. This point leads surgeons to performing hemi-PETS with a sufficient period of remaining growth. As a result, surgical outcomes of hemi-PETS are generally satisfactory but change after removal of screws until skeletal maturation [8,9,10,11,12].

Occasionally, surgeons first meet patients with angular deformity near skeletal maturation. Compared with tension-band plating, one of other recent options for hemiepiphysiodesis, the correction speed of hemi-PETS was faster [16,17]. Thus, hemi-PETS, rather than tension-band plating, may be a better option for angular correction in these patients considering the advantages resulting from less invasiveness and faster correction speed. Unlike other options, the possibility of physeal damage is raised after hemi-PETS as the screws directly penetrate growth plates [8,11]. However, it is not an important issue in patients very near skeletal maturation who are concerned about undercorrection. It seems that the ideal time frame for surgery, rather than the choice of surgical option, should be studied further in patients near skeletal maturation. However, the upper age limit for hemi-PETS to correct angular deformity (i.e., the latest timing for surgery resulting in sufficient correction) is unclear and has not been studied yet. Reviewing surgical outcomes, including undercorrection in patients near skeletal maturation, should be required.

With this background, the purpose of this study was to review postoperative courses after hemi-PETS for genu varum. We focused on the degree of skeletal maturation leading to undercorrection to suggest the upper age limit for hemi-PETS in terms of correcting genu varum. In addition to the traditional method of assessing bone age (BA) based on radiographs of the hand in the atlas [18], which is relatively complex for rapid application in a busy clinical setting [19], we included the condition of epiphyseal fusion of distal phalanges in the hand as a practical indicator for simplified clinical use. The epiphyseal fusion of various hand bones has a certain sequence. The distal phalangeal physes are fused at about 1 year before the end of skeletal maturation, and then the proximal and middle phalangeal physes are fused in order [18,19]. We hypothesized that hemi-PETS performed after epiphyseal fusion of distal phalanges in the hand would result in undercorrection of genu varum.

## 2. Materials and Methods

### 2.1. Selection of the Study Subjects

The Institutional Review Board of Samsung Medical Center approved the study protocol. The medical records of adolescents who underwent hemi-PETS to correct genu varum between 2020 and 2022 at Samsung Medical Center were retrospectively reviewed. Among various methods of hemiepiphysiodesis, we exclusively performed hemi-PETS on all adolescents with idiopathic genu varum during the study period. The exclusion criteria were as follows: (1) genu varum < 5° based on the hip–knee–ankle (HKA) alignment (as this study focused on undercorrection after surgery, patients with minor deformities were excluded; as a result, 3 patients were excluded); (2) hemi-PETS performed also at the distal femur (to construct study subjects with a homogeneity of surgical procedures, 2 patients were excluded); (3) no hand radiograph within 2 weeks from surgery (2 patients); and (4) not followed-up to the completion of skeletal maturation (1 patient). As a result, the selected 29 patients had no specific reasons for genu varum and underwent hemi-PETS only at the proximal tibia. Considering the principle of statistical independence [20], one side was randomly selected in bilateral cases using a program that is freely available (http://www.randomizer.org (accessed on 1 June 2024)).

### 2.2. Surgical Technique

**Figure 1:**
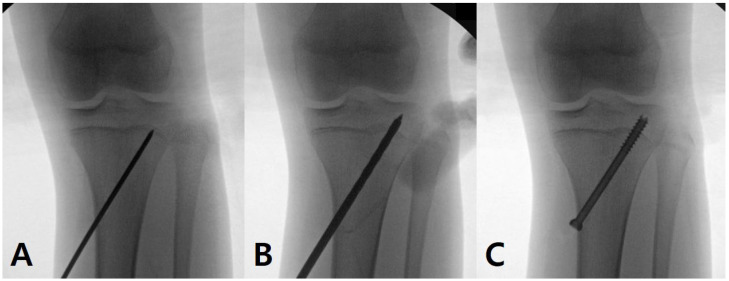
Hemi-percutaneous epiphysiodesis using a transphyseal screw at the lateral portion of the proximal tibial physis. A 6.5 mm cannulated screw was inserted through a 1.0 cm skin incision from medial proximal metaphysis. The lateral one fourth of the physis was targeted using a guide pin. To minimize the physeal damage, the guide pin entered the epiphysis after confirming its position by intraoperative radiographs (**A**). During drilling, continuous saline irrigation was performed and a cannulated drill bit repeatedly changed into the other one to minimize the thermal damage (**B**). The final screw position had enough threads (at least three) on both the below and top of the physis (**C**).

**Figure 2:**
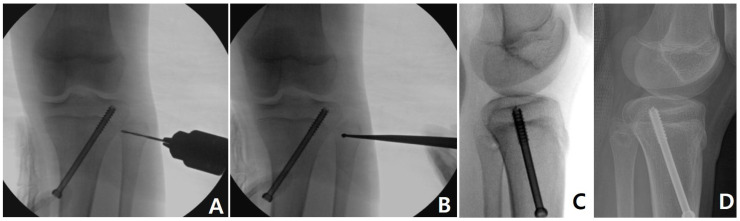
Percutaneous epiphysiodesis of the proximal fibular physis. Through a 0.8 cm skin incision, serial drilling using 2.0~4.4 mm drill bits (**A**) and additional curettage (**B**) were performed. Intraoperative (**C**) and postoperative (**D**) lateral radiographs showed related defects after the procedures.

Considering the size of epiphysis, a 6.5 or 7.3 mm cannulated screw was selected and percutaneously inserted (Figure 1). As a combined procedure, percutaneous epiphysiodesis [21] (Figure 2) was performed at the proximal fibular physis when the remaining growth in the proximal tibial physis was expected to be >1.0 cm [22] or the patient complained about the cosmetic prominence of the fibula head. As a result, it was performed in 22 cases (75.9%). The screw was removed after confirming the alignment between a neutral alignment (HKA alignment of 0°) and overcorrection of the 1.0° (valgus 1.0°) while considering the rebound phenomenon [8,11]. If the degree of correction was not sufficient, the screw was kept until the completion of skeletal maturation.

### 2.3. Radiographic Measurement

A standing full-length anteroposterior (AP) radiograph of the lower limbs was periodically taken and used to assess the alignment of the lower limb. The radiograph was checked with both knees fully extended and with both patellae facing forwards to minimize measurement errors. The HKA alignment was defined as an alignment between the mechanical axis of the femur and that of the tibia. The medial proximal tibial angle (MPTA) was measured to describe the alignment of the proximal tibia [23]. Between the two indicators, the HKA alignment was used to analyze surgical outcomes because it was more reliable than the MPTA based on their results of intraclass correlation coefficients (ICCs, presented in Table 1). The final correction angle obtained by surgery (f-CA, HKA alignment at the time of hemi-PETS—final HKA alignment) and the magnitude of rebound phenomenon (rebound angle, final HKA alignment—HKA alignment at the time of screw removal) were calculated. To define the acceptable correction, we used two criteria. Considering the degree of rebound phenomenon after hemi-PETS at the proximal tibia (approximately 1.0°) [8,11], the first criterion of the final HKA alignment (<varus 1.0°) was set. Surgical results in some patients can be evaluated as undercorrection even after significant postoperative corrections due to their greater preoperative deformity. Thus, the second criterion of the f-CA (>6.5°) was set while considering the distribution of preoperative HKA alignment in our cohort (varus 6.5 ± 1.3°). In summary, the acceptable correction was defined as (1) the final HKA alignment < varus 1.0° or (2) the f-CA > 6.5°.

**Figure 3:**
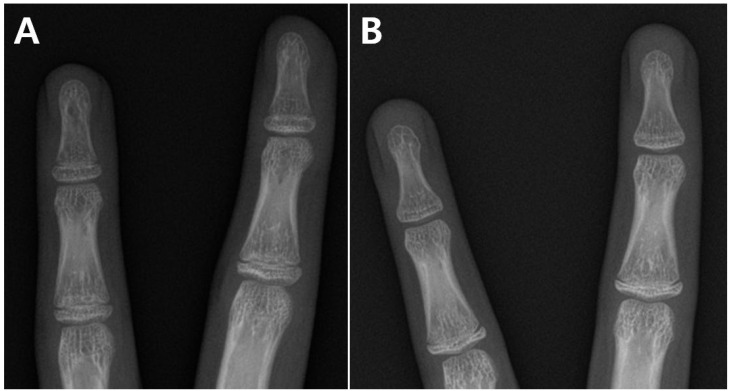
Examples of epiphyseal fusion of distal phalanges in the hand. The 3rd and 4th fingers in two patients of the fusion group. Two distal phalanges were subclassified as partial fusion (**A**) and the other two were subclassified as complete fusion (**B**). The reason for performing surgery in this patient was that the epiphyseal fusion of the middle phalanges was not complete (**B**).

The study subjects were considered as candidates for hem-PETS. Thus, a radiograph of the hand and wrist was obtained periodically to determine the timing for surgery. The BA was estimated based on a radiological atlas [18]. The period of remaining growth was calculated using the BA with the assumption that growth around the knee ends at 16 years in boys and 14 years in girls [24], which were defined as the respective ends of skeletal maturation in this study. If the finding did not fit both adjacent standards of the atlas [18], the mean value of the two standards was used as the BA [8]. We additionally evaluated the condition of the epiphyseal fusion of distal phalanges in the hand and classified it as open, partial fusion or complete fusion. If the epiphysis of distal phalanges had not begun to fuse with its shaft (i.e., the radiolucent space between the epiphysis and shaft was identified as continuous even if thin), the condition was classified as open. If the epiphysis had begun to fuse with its shaft but the radiolucent space did not completely disappear, the condition was classified as a partial fusion. If the radiolucent space between the epiphysis and shaft completely disappeared and their radial and ulnar margins were connected, the condition was classified as complete fusion (Figure 3). If patients had 5 open distal phalanges, they were classified as the open group (OG). If at least one epiphysis had begun to fuse (partial or complete fusion), the patients were classified as the fusion group (FG).

**Figure 4:**
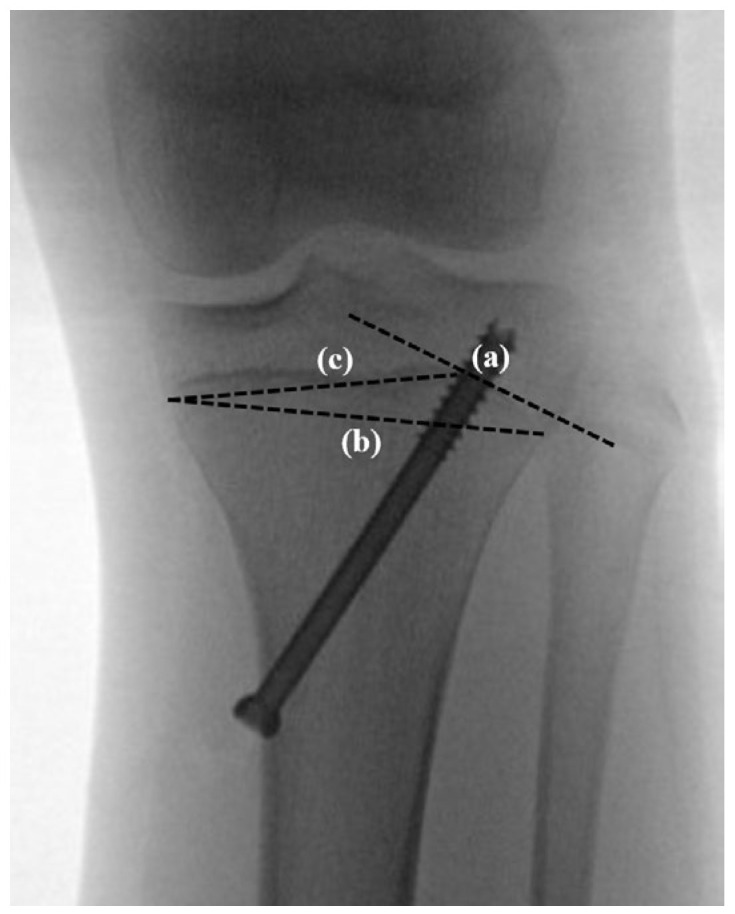
Indicators related to surgical techniques. A tangent line to the physis at the point where screw threads penetrate the physis (**a**) was drawn and used to measure the angle with the screw. The moment arm of the screw (%) was calculated (length of line c/length of line b × 100) based on the physis at the point just medial to the screw. (**b**) A line connecting both ends of the physis. (**c**) A line connecting the physis at the point just medial to the screw and its medial end.

Regarding indicators related to surgical techniques, a knee AP radiograph was used because genu varum is an angular deformity in the coronal plane. The angle between the screw and the physis (screw angle, SA), which was reported to be positively associated with the efficacy in PETS [15], was measured using a tangent line to the physis at the point where the screw threads penetrate the physis [11]. The moment arm of the screw (moment arm, MA) was calculated based on the physis at the point just medial to the screw [11] (Figure 4).

All radiographic measurements were performed using the PACS system (GE Healthcare, Chicago, IL, USA). All indicators were measured twice (at intervals of, at minimum, 2 weeks) by two observers, and the calculated mean values (for the HKA alignment, MPTA, BA, SA and MA) and the agreed results (classification of epiphyseal fusion, if different) were used. The reliabilities regarding the HKA alignment, MPTA, BA, SA and MA, assessed by calculating the ICCs, ranged from excellent to good [25]. Details of the ICCs are presented in Table 1. The Cohen’s kappa for classification of epiphyseal fusion between two observers was calculated as 0.84 and almost perfect [26].

### 2.4. Statistical Analysis

All numerical data are presented as mean ± standard deviation to one decimal place. A Fisher’s exact test and a Mann–Whitney U test were used to compare the OG and FG while considering the number of study subjects. We designed two linear regression models to describe the significant factors for the f-CA. Considering the number of study subjects (n = 29), each model used 5 independent variables. The (1) HKA alignment before surgery, (2) SA, and (3) MA were equally used in the two models. In addition, the first model included the (4) sex and (5) period of remaining growth. The second used the occurrence of (4) partial and (5) complete fusion of distal phalanges. R ver. 4.2.0 (R Foundation for Statistical Computing, Vienna, Austria) was used with the significance level set at *p* < 0.05.

## 3. Results

### 3.1. Comparisons Between the Open and Fusion Groups

In our cohort, there were 12 males (aged 13.4 ± 0.5) and 17 females (aged 11.8 ± 0.6). The period of remaining growth was 1.8 ± 0.5 in males and 1.8 ± 0.7 in females at the time of hemi-PETS. In the selected 29 lower limbs, the mean HKA alignment was varus 6.5 ± 1.3° at the time of hemi-PETS. The screws were kept for 602.6 ± 241.6 days and then removed. The mean f-CA was 5.8 ± 2.0° with a rebound angle of 0.3 ± 1.0°. There were no complications related to surgical procedures, such as neurovascular injury or infection.

Ten patients indicated the partial or complete fusion of the epiphysis of distal phalanges in the hand at the time of hemi-PETS and were classified as the FG. The mean f-CA of the FG was 4.0 ± 1.9°, which was significantly smaller than that (6.7 ± 1.3°, *p* = 0.001) of the OG. The post hoc statistical power of this comparison between the two groups was 98.1% with a = 0.05. The acceptable correction was obtained in all 19 patients of the OG. In the OG, the period of remaining growth ranged 2.0~2.5 years in six males and 1.5~2.5 years in nine females. Otherwise, the acceptable correction was obtained in just two patients in the FG (19/19 vs. 2/10, *p* < 0.001). The period of remaining growth ranged 11~18 months in six males and 3~12 months in four females in the FG. Two indicators related to surgical techniques (the SA and MA) were not significantly different between the two groups. Details of the two groups and their comparisons are presented in Table 2.

### 3.2. Details of the Fusion Group

Among the 10 patients in the FG, two patients preoperatively showed complete fusion of the epiphysis of distal phalanges in the hand (patients 6 and 10 in Table 3). Their f-CAs were 0.7 and 1.1°, indicating substantial undercorrection (9.2 and 14.5%, dividing the f-CA by the preoperative HKA alignment). Except for the two patients with complete fusion, the f-CA ranged 3.3~6.4° (51.5~100%) in the remaining eight patients without complete fusion in the FG. In particular, 3 patients had 5 epiphyses with partial fusion (patients 5, 8, and 9 in Table 3). Their period of remaining growth was 16 months in a male and 10~11 months in two females. It is noteworthy that two of these three patients were the cases of acceptable correction in the FG. The detailed data of the FG are presented in Table 3.

### 3.3. Linear Regression Analyses to Describe the f-CA

In the two linear regression models, the preoperative HKA alignment showed the statistical significance. On the other hand, two indicators related to surgical techniques were not statistically significant for the f-CA. Independent variables related to the degree of skeletal maturation (sex and period of remaining growth in Model 1, partial and complete fusion of distal phalanges in Model 2) showed the statistical significance in the two models. Model 2 showed a higher R^2^ value and lower AIC value compared to Model 1. In other words, the condition of the epiphyseal fusion of distal phalanges was better at describing the variation in the f-CA compared to traditional indicators, the sex and the period of remaining growth in our cohort. The details of the two linear regression models are presented in Table 4.

## 4. Discussion

The most important finding of this study was that the condition of epiphyseal fusion of distal phalanges in the hand can be used as a practical indicator of undercorrection after hemi-PETS at the proximal tibia in regard to genu varum. Considering that screws were removed about 6 months after hemi-PETS performed at both the proximal tibia and distal femur in patients with a sufficient period of remaining growth [8,11], a remaining growth period of less than 1 year after hemi-PETS at the proximal tibia exclusively is expected to be insufficient. The distal phalangeal physes in the hand are fused about 1 year before the end of skeletal maturation [18,19]. Thus, we included its condition in this study. First, all 19 patients who underwent surgery before the beginning of epiphyseal fusion of distal phalanges showed acceptable corrections. Second, eight patients who underwent surgery before complete epiphyseal fusion but after partial fusion showed the partial effects of 3.3~6.4° (51.5~100% of deformity). Their period of remaining growth ranged 16~18 months in males and 10~12 months in females. Third, two patients who underwent surgery after complete epiphyseal fusion showed the limited effects of 0.7 and 1.1°. Based on these results, we suggest the upper part of the period of remaining growth in the second group (18 months in males and 12 months in females) as the upper age limit for hemi-PETS at the proximal tibia in terms of correcting genu varum in adolescent patients. Of course, performing the surgery before the beginning of epiphyseal fusion of distal phalanges would be better in terms of minimizing the possibility of undercorrection. In contrast to previous studies that reported the efficacy of hemiepiphysiodesis around the knee in growing patients [6,7,8,9,10,11,12], we included patients near skeletal maturation and analyzed the upper age limit in regard to performing the surgery. This point is the unique element and strength of our study.

The distal femoral and proximal tibial physes are feasible targets for hemiepiphysiodesis to correct angular deformities of the knee because they are close to the center of the deformities and account for the majority of the growth of the lower limb [22]. To achieve anatomical correction of angular deformity, it is essential to accurately determine what anatomical structures caused the deformity. If there is only one deformed site between the two targets, only correcting the deformed site would be ideal. In the 29 lower limbs with preoperative deformity of varus 6.5 ± 1.3°, the mean preoperative MPTA was 83.0 ± 1.5°. Considering the reference rage of the MPTA (87.5 ± 1.5°) [27,28,29], it seems that the predominant site of deformity was the proximal tibia rather than the distal femur. The predominant deformity at the proximal tibia suggests the need for surgery only at the proximal tibia when considering obtaining anatomical correction. As a result, our cohort underwent hemi-PETS only at the proximal tibia. During the study period, we performed hemi-PETS at both the distal femur and proximal tibia in two patients who were excluded due to the homogeneity of the study subjects. There were no patients who only underwent hemi-PETS at the distal femur during the period. If hemi-PETS was performed at both the proximal tibia and distal femur, the f-CA would be greater in some patients in the FG due to the amount of remaining growth at the distal femur being relatively greater than that in the proximal tibia in growing patients [22]. In other words, performing hemi-PETS at both the proximal tibia and distal femur can be a feasible surgical option in patients with an uncertain period of remaining growth in regard to obtaining sufficient corrections. Future studies should focus on this point. Consequently, our suggestion of the upper age limit is difficult to apply to genu valgum that is mainly corrected by hemiepiphysiodesis at both the distal femur and proximal tibia [8,11].

Two patients with a complete epiphyseal fusion of distal phalanges in the hand (patients 6 and 10 in Table 3) showed a partial epiphyseal fusion of the middle phalanges. Although their skeletal maturation was near completion, because they were not considered to be completed, the two underwent surgery after agreeing on the possibility of undercorrection. By reviewing the range of the period of remaining growth in the eight patients who underwent surgery before complete epiphyseal fusion but after partial fusion, the extent of the range was just 2 months (16~18 months in males and 10~12 months in females). The BAs of adjacent standards in the atlas have a difference of several months [18]. This study was motivated by the need for more practical indicators of simplified clinical use in surgical planning. In our linear regression analyses, Model 2, which used the condition of the epiphyseal fusion of distal phalanges, showed a higher R^2^ value and lower AIC value compared to Model 1, which used the traditional BA. These findings also suggest that the condition of epiphyseal fusion can be used as a practical indicator of undercorrection after hemi-PETS for genu varum instead of the traditional BA.

There were several limitations to this study, particularly due to its retrospective design. This study was also limited by the possibility of bias and type II errors because of the small number of subjects. The effect of the epiphysiodesis of the proximal fibular physis was also a limitation. However, it was skipped in seven patients near skeletal maturation. Thus, its effect on their final alignment and the following surgical results would be minimal. Lastly, the BA was evaluated based only on a hand radiograph. There might be a difference compared with actual growth around the knee [30].

## 5. Conclusions

In conclusion, the condition of epiphyseal fusion of distal phalanges in the hand can be used for simplified clinical use in surgical planning of hemi-PETS at the proximal tibia to correct genu varum. To minimize the possibility of undercorrection, the surgery should be performed before the beginning of the epiphyseal fusion of distal phalanges. We suggest a period of remaining growth of 18 months in males and 12 months in females as being the upper age limit for the surgery. After complete epiphyseal fusion of distal phalanges, performing hemi-PETS exclusively at the proximal tibia should be avoided.

## Figures and Tables

**Table 1:** Intraclass correlation coefficients of intra- and interobserver differences in measuring radiographic indicators.

	Observer 1 *	Observer 2 *	Interobserver ^†^
HKA alignment (°)	0.91	0.94	0.92
MPTA (°)	0.82	0.85	0.81
Bone age (years)	0.84	0.89	0.83
Screw angle (°)	0.85	0.82	0.81
Moment arm of the screw (%)	0.88	0.87	0.85

HKA, hip–knee–ankle; MPTA, medial proximal tibial angle. * Calculated using the two measured values in each observer. ^†^ Calculated using the mean of two measurements in observer 1 and that in observer 2.

**Table 2:** Details of comparisons between the open (n = 19) and fusion (n = 10) groups.

Variables	Open Group (n = 19)	Fusion Group (n = 10)	*p*	*Cohen’s d*
Sex (male–female)	6 (31.6%):13 (68.4%)	6 (60.0%):4 (40.0%)	0.236 *	N/A
Bone age (years) ^a^	12.5 ± 0.9	14.1 ± 0.8	0.001 ^†^	1.879
Remaining growth (years) ^a^	2.1 ± 0.3	1.1 ± 0.4	<0.001 ^†^	2.828
Body mass index (kg/m^2^) ^a^	17.5 ± 1.7	18.9 ± 2.7	0.161 ^†^	0.621
Side (right–left)	10 (52.6%):9 (47.4%)	4 (40.0%):6 (60.0%)	0.700 *	N/A
HKA alignment (°) ^a^	Varus 6.3 ± 1.2	Varus 6.9 ± 1.4	0.232 ^†^	0.460
MPTA (°) ^a^	83.3 ± 1.3	82.4 ± 1.7	0.301 ^†^	0.595
Screw angle (°) ^a^	79.7 ± 5.4	81.0 ± 5.0	0.581 ^†^	0.250
Moment arm of the screw (%) ^a^	76.9 ± 2.8	77.0 ± 2.9	0.945 ^†^	0.035
Final correction angle (°)	6.7 ± 1.3	4.0 ± 1.9	0.001 ^†^	1.659
Rebound angle (°)	0.3 ± 1.2	0.3 ± 0.7	0.909 ^†^	0.000
Acceptable correction (%)	19/19 (100%)	2/10 (20.0%)	<0.001 *	N/A

HKA, hip–knee–ankle; MPTA, medial proximal tibial angle; N/A, not applicable. ^a^ At the time of hemi-percutaneous epiphysiodesis using transphyseal screws. * Obtained by using Fisher’s exact tests. ^†^ Obtained by using Mann–Whitney U tests.

**Table 3:** Detailed data of the fusion group (n = 10).

No.	Sex	Remaining Growth ^a,b^	Open ^c^	Partial Fusion ^c^	Complete Fusion ^c^	HKA Alignment ^b^	f-CA ^d^	Acceptable Correction
1	Male	1^+6^ year	2	3	0	5.5°	4.3° (78.2%)	No
2	Male	1^+6^ year	2	3	0	6.3°	3.9° (61.9%)	No
3	Male	1^+6^ year	2	3	0	9.3°	4.9° (52.7%)	No
4	Male	1^+5^ year	1	4	0	5.6°	3.3° (58.9%)	No
5	Male	1^+4^ year	0	5	0	5.4°	5.3° (98.1%)	Yes
6	Male	11 months	0	2	3	7.6°	0.7° (9.2%)	No
7	Female	1 year	2	3	0	9.0°	6.3° (70.0%)	No
8	Female	11 months	0	5	0	6.2°	6.4° (100%)	Yes
9	Female	10 months	0	5	0	6.6°	3.4° (51.5%)	No
10	Female	3 months	0	0	5	7.6°	1.1° (14.5%)	No

HKA, hip–knee–ankle; f-CA, final correction angle obtained by surgery. ^a^ Presented in descending order in each sex. Notably, 1 year and X months was described as 1^+X^ year for simplicity. ^b^ At the time of hemi-percutaneous epiphysiodesis using transphyseal screws. ^c^ The number of corresponding epiphyses of distal phalanges in a hand. ^d^ Percentage (%) was calculated as f-CA°/HKA alignment° × 100. If > 100, it was presented as 100.

**Table 4:** Details of two linear regression models setting the final correction angle as a dependent variable (n = 29).

	Model 1		Model 2	
	**Adjusted Coefficient (95% CI)**	***p***	**Adjusted Coefficient (95% CI)**	***p***
HKA alignment (°) ^a^	0.44 (0.06, 0.82)	0.033	0.53 (0.22, 0.85)	0.003
Screw angle (°) ^a^	0 (−0.1, 0.11)	0.958	0 (−0.09, 0.08)	0.97
Moment arm of the screw (%) ^a^	−0.1 (−0.29, 0.08)	0.29	−0.11 (−0.27, 0.04)	0.169
Sex ^b^	1.16 (0.16, 2.15)	0.033	N/A	
Remaining growth (years) ^a^	2.67 (1.79, 3.54)	<0.001	N/A	
Partial fusion ^a,c^	N/A		−2.15 (−3.03, −1.27)	<0.001
Complete fusion ^a,c,d^	N/A		−4.55 (−6.33, −2.76)	<0.001
	R^2^ = 0.670		R^2^ = 0.777	
	AIC value = 103.7		AIC value = 92.2	

CI, confidence interval; HKA, hip–knee–ankle; N/A, not applicable. ^a^ At the time of hemi-percutaneous epiphysiodesis using transphyseal screws. ^b^ Coded as male = 0, female = 1. ^c^ Epiphyseal fusion of distal phalanges in the hand (coded as no = 0, yes =1). ^d^ If coded as 1, partial fusion was also coded as 1.

## Data Availability

The datasets analyzed during the current study are available from the corresponding author upon reasonable request.
